# Using Xbox kinect motion capture technology to improve clinical rehabilitation outcomes for balance and cardiovascular health in an individual with chronic TBI

**DOI:** 10.1186/s40945-017-0033-9

**Published:** 2017-05-31

**Authors:** Shane Chanpimol, Bryant Seamon, Haniel Hernandez, Michael Harris-Love, Marc R. Blackman

**Affiliations:** 1 Neurology Service, Veterans Affairs Medical Center, Washington, DC USA; 2 Physical Medicine and Rehabilitation Service, Veterans Affairs Medical Center, Washington, DC USA; 3 Muscle Morphology, Mechanics and Performance Laboratory, Clinical Research Center - Human Performance Research Unit, Veterans Affairs Medical Center, Washington, DC USA; 4 Geriatrics and Extended Care Service/Research Service, Veterans Affairs Medical Center, Washington, DC USA; 5 Department of Exercise and Nutritional Sciences, Milken Institute School of Public Health, The George Washington University, Washington, DC USA; 6 Research Service, Veterans Affairs Medical Center, Washington, DC USA; 7 Departments of Medicine, Biochemistry and Molecular Medicine, George Washington University School of Medicine, Washington, DC USA; 8 Departments of Medicine and Rehabilitation Medicine, Georgetown University School of Medicine, Washington, DC USA

**Keywords:** Virtual reality, Xbox kinect, Physical therapy, Intervention, Traumatic brain injury

## Abstract

**Background:**

Motion capture virtual reality-based rehabilitation has become more common. However, therapists face challenges to the implementation of virtual reality (VR) in clinical settings. Use of motion capture technology such as the Xbox Kinect may provide a useful rehabilitation tool for the treatment of postural instability and cardiovascular deconditioning in individuals with chronic severe traumatic brain injury (TBI). The primary purpose of this study was to evaluate the effects of a Kinect-based VR intervention using commercially available motion capture games on balance outcomes for an individual with chronic TBI. The secondary purpose was to assess the feasibility of this intervention for eliciting cardiovascular adaptations.

**Methods:**

A single system experimental design (*n* = 1) was utilized, which included baseline, intervention, and retention phases. Repeated measures were used to evaluate the effects of an 8-week supervised exercise intervention using two Xbox One Kinect games. Balance was characterized using the dynamic gait index (DGI), functional reach test (FRT), and Limits of Stability (LOS) test on the NeuroCom Balance Master. The LOS assesses end-point excursion (EPE), maximal excursion (MXE), and directional control (DCL) during weight-shifting tasks. Cardiovascular and activity measures were characterized by heart rate at the end of exercise (HRe), total gameplay time (TAT), and time spent in a therapeutic heart rate (TTR) during the Kinect intervention. Chi-square and ANOVA testing were used to analyze the data.

**Results:**

Dynamic balance, characterized by the DGI, increased during the intervention phase *χ* ^2^ (1, *N =* 12) = 12, *p* = .001. Static balance, characterized by the FRT showed no significant changes. The EPE increased during the intervention phase in the backward direction *χ* ^2^ (1, *N =* 12) = 5.6, *p* = .02, and notable improvements of DCL were demonstrated in all directions. HRe (*F* (2,174) = 29.65, *p* = < .001) and time in a TTR (*F* (2, 12) = 4.19, *p* = .04) decreased over the course of the intervention phase.

**Conclusions:**

Use of a supervised Kinect-based program that incorporated commercial games improved dynamic balance for an individual post severe TBI. Additionally, moderate cardiovascular activity was achieved through motion capture gaming. Further studies appear warranted to determine the potential therapeutic utility of commercial VR games in this patient population.

**Trial registration:**

Clinicaltrial.gov ID - NCT02889289

## Background

The last two decades demonstrated an exponential trend in the implementation of virtual reality (VR) in clinical settings [1]. Researchers and clinicians alike are enticed by the potential of this technology to enhance neuroplasticity secondary to rehabilitation interventions. Currently, Nintendo Wii, Sony PlayStation, and Microsoft Xbox offer commercially developed semi-immersive VR platforms which are used for rehabilitation [2]. Several studies report positive effects of these commercial technologies for improving balance, coordination and strength [3–5]. In 2010, Microsoft introduced a novel infrared camera that works on the Xbox platform called Kinect. The Kinect camera replaces hand held remote controls through the use of whole body motion capture technology.

Whole body motion capture VR allows a unique opportunity for individuals to experience a heightened sense of realism during task-specific therapeutic activities. However, clinicians need to be able to match a game’s components to an individual’s functional deficits. Seamon et al. [6] provided a clinical demonstration of how the Kinect platform can be used with Gentiles taxonomy for progressively challenging postural stability and influencing motor learning in a patient with progressive supranuclear palsy. Similarly, Levac et al. [7] developed a clinical framework titled, “Kinecting with Clinicians” (KWiC) to broadly address implementation barriers. The KWiC resource describes mini-games from Kinect Adventures on the Xbox 360 in order to provide a comprehensive document for clinicians to reference. Clinicians can use KWiC to base game selection and play on their client’s goals and the therapist’s plan of care for that individual.

In parallel with knowledge translation research, several studies found postural control improvements in multiple diagnostic groups including individuals with chronic stroke [8–10], Friedrich’s Ataxia [11], multiple sclerosis [12], Parkinson’s disease [13], and mild to moderate traumatic brain injury (TBI) [14] when using Kinect based rehabilitation. Additional research shows that exercising with the Kinect system can reach an appropriate intensity for cardiovascular adaptation. For example, Neves et al. [15] and Salonini et al. [16] reported increases in exercise heart rate and blood pressure in healthy individuals and children with cystic fibrosis while playing Kinect games. Similarly, Kafri et al. [17] reported the ability of individuals post-stroke to reach levels of light to moderate intensity using Kinect games.

Individuals with TBI are likely to have a peak aerobic capacity 65–74% to that of healthy control subjects [18]. There is limited research on cardiovascular training after severe TBI [18]. However, Bateman et al. [19] demonstrated that individuals with severe TBI can improve cardiovascular fitness during a 12-week program participants exercised at an intensity equal to 60–80% of their maximum heart rate 3 days per week. Commercial Xbox Kinect games, such as Just Dance 3, have been shown to improve cardiovascular outcomes for individuals with chronic stroke [20]. However, there is a lack of research investigating the efficacy of motion capture VR on cardiovascular health for individuals with chronic severe TBI. Walker et al. [21] makes the recommendation for rehabilitation programs to go beyond independence in basic mobility and to develop treatment strategies to address high-level physical activities. The high rates of sedentary behavior in individuals across all severities of TBI could be attributed the lack of addressing these limitations in activity.

Postural instability is the second most frequent, self-reported limitation, 5 years post injury for individuals with severe TBI [22]. It is unknown whether use of motion capture VR in individuals with severe, chronic TBI can address neuromotor impairments related to high-level activities such as maintaining postural control during walking. Similarly, there is a need to determine if training with VR motion capture can attain necessary intensity levels for inducing cardiovascular adaptation. Due to this knowledge gap and heterogencity of individuals post TBI, feasibility of investigatory interventions should be explored prior to examining effectiveness with randomized control trials. Single system experimental design (SSED) provides a higher level of rigor compared to case studies based on the ability to compare outcomes across phase conditions with the participant acting as their own control. The value of SSED within rehabilitation has been noted by other investigators [23, 24] making it an attractive design for practitioners aiming to gain insight into novel clinical interventions prior to large scale clinical trials. The purpose of this proof of concept and feasibility study was to evaluate the effectiveness of commercially available Xbox One Kinect games as a treatment modality for the rehabilitation of balance and cardiovascular fitness for a veteran with chronic severe TBI. Additionally, we provide herein a description of the Kinect games to assist providers with clinical implementation.

## Methods

A single system experimental design was used to evaluate the effects of supervised physical therapy sessions on clinical outcomes of balance and cardiovascular fitness using commercially available games on the Xbox One with Kinect System. The study design included a baseline, intervention, and retention phase (Table 1). The baseline phase included 7 balance assessments over 12 weeks. The intervention phase included cardiovascular outcomes from each intervention session and 5 balance assessments over 8 weeks. Finally, the retention period included 5 balance assessments over 4 weeks. All repeated measures were taken by an experienced physical therapist who was masked to intervention treatment status. The study was conducted between May and November of 2015 at the Washington DC Veterans Affairs Medical Center and approved by its Institutional Review Board and Research and Development Committee.

**Table 1:** Study timeline and assessment schedule

Phase (weeks)	Week	Gameplay/Activity Assessment	Balance Assessment
Baseline	0–13	0	7, 1 assessment per 2 weeks
Intervention	14–21	15, 2 sessions per week	5, 1 assessment per 1.5 weeks
Retention	22–26	0	5, 1 assessment per 1.5 weeks

Male or female Veterans between the ages of 18 and 65 years old that had sustained a moderate to severe TBI greater than 1 year prior to baseline assessments were eligible to be included. Veterans were excluded if they had: any cardiac condition that may have caused sudden decompensation during cardiovascular testing/training (e.g. CHF, uncontrolled hypertension), a history of behavioral impairments (e.g. aggression or inappropriate actions) that would preclude participation in a physical therapy setting, lower extremity amputation, moderate or severe cognitive impairment (score less than 17/30 on the Montreal Cognitive Assessment). Veterans were also excluded if they were unable to stand unsupported for at least 2 min, and unable to ambulate on a treadmill with bilateral hand support. Potential participants were informed of the study and referred by their Physical Medicine and Rehabilitation physician for recruitment.

### Participant

The study participant was a 37-year-old male Caucasian Air Force veteran. He sustained a closed head injury and resultant severe TBI (initial Glasgow Coma Scale (GCS) 5) resultant from a motor vehicle accident in 2004. He began acute rehabilitation one month post injury (GCS 11) at Walter Reed Naval National Medical Center. After discharge from inpatient rehabilitation he participated in outpatient therapy including physical, occupational, speech-pathology, and recreation therapy with the Department of Veterans Affairs under the Polytrauma System of Care.

Before the study, the participant lived independently with his wife. He was able to independently manage his schedule and transportation to appointments. His self-reported primary deficits were balance instability and fatigue when interviewed prior to the intervention. The participant did not require an assistive device for ambulation. Qualitative observation of his gait revealed a wide base of support and high-steppage pattern. Table 2 provides further details regarding the participant’s baseline balance function. During the study, he took an ACE inhibitor (Lisinopril 20 mg once daily) and beta-blocker (Metoprolol tartrate 50 mg twice daily) to control high blood pressure. No alterations to the participant’s medications were made during the study. He was an active smoker, smoking ¼ of a pack per day.

**Table 2:** Mean scores of repeated measures for balance outcomes by phase

		Phase	
Outcome	Baseline (SD)	Intervention (SD)	Retention (SD)
DGI	11.8 (0.4)	16.2 (2.3)	19 (0.0)
FRT (cm)			
Both	28.9 (2.9)	28.9 (1.9)	26.9 (1.6)
Left	37.0 (2.3)	36.8 (1.0)	34.7 (1.4)
Right	36.6 (1.9)	37.5 (2.0)	36.0 (2.0)
EPE (%)			
Front	67.3 (10.2)	69.9 (6.5)	80.2 (10.5)
Right	69.4 (6.1)	70.5 (8.6)	70.0 (2.9)
Back	74.1 (5.4)	84.6 (13.0)	78.1 (9.6)
Left	75.0 (5.7)	79.3 (12.1)	73.5 (6.8)
MXE (%)			
Front	98.5 (8.6)	99.7 (5.3)	107.0 (3.8)
Right	94.2 (6.9)	98.7 (6.3)	100.4 (2.9)
Back	100.1 (7.6)	108.3 (7.2)	106.6 (10.7)
Left	99.8 (8.5)	101.8 (7.8)	107.0 (6.7)
DCL (%)			
Front	67.33 (5.8)	74.2 (4.8)	67.5 (7.5)
Right	55.02 (11.3)	66.8 (8.6)	64.2 (9.4)
Back	50.5 (8.6)	59.1 (3.0)	60.5 (10.4)
Left	68.3 (4.3)	73.1 (3.6)	68.5 (4.5)

*DGI* dynamic gait index, *FRT* functional reach test *EPE* end-point excursion, *MXE* maximal excursion, *DCL* directional control

The participant reported via interview that he had a sedentary lifestyle with the exception of regular leisure activity with the Washington DC VAMC recreation therapy service. This included a kayaking skill building course, social outings, and individual recreation therapy to promote engagement in leisure activity which occurred 1–3 times per week. The participant was instructed to maintain his ongoing recreation and leisure activities throughout the study. During the study period there were no significant changes to his participation in recreation therapy services.

### Measurements

Repeated measures of all outcomes related to balance and cardiovascular health were collected during each study phase by a board certified neurologic physical therapist who was blinded to the intervention. The order of assessments is presented in Table 1.

#### Balance

Measures used to assess balance included the Dynamic Gait Index (DGI), Functional Reach Test (FRT), and the Limits of stability (LOS) test on the Neurocom Balance Master. The DGI is a clinical measure that evaluates an individual’s balance while performing tasks with a moving base of support (BOS). The DGI is able to detect small, but clinically relevant, changes in fall risk in patients with TBI. [25] Three subtests of the DGI have been found to have significant correlations with subjective complaints of balance impairments in patients with mTBI. These include “gait with vertical head turns”, “gait with horizontal head turns” and “gait and pivot turn”. [26] The FRT is a clinical measure used to evaluate an individual’s ability to displace their center of gravity (COG) forward without losing their balance. No psychometric properties have been specifically established for TBI. However, excellent reliability and high validity was shown in individuals with subacute stroke by Outermans et al. [27]. A cut-off score <15 cm for increased fall risk post stroke has been established as well [28]. The FRT was taken under three conditions: reaching forward with palms together (FRTb), right hand only (FRTr), and left hand only (FRTl).

The LOS is utilized to characterize static balance in all directions. The LOS is comprised of 8 trials in which the individual must displace their COG toward the front, right, left, back, and between directions. Each trial begins with a period of static stance in which the subject can visualize their COG. The Neurocom then prompts the individual to move their COG toward a specified target in 1 of the 8 directions. Neurocom records several aspects of postural control which include end-point excursion (EPE), maximum excursion (MXE), and directional control (DCL). EPE evaluated the distance the participant’s COG moved in their first attempt to reach the target. The MXE demonstrated the farthest distance the COG moved. Both the EPE and MXE are scored as percentages of expected excursions for an individual’s height. DCL measures the accuracy of the COG moving toward the target. The three individual measures within the LOS were averaged into front (LOSf), right (LOSr), back (LOSb), and left (LOSl) categories. For example, the 3 forward trials were averaged to create one ‘front’ average for each data collection point. Definitive validity and reliability have not been established for the Neurocom LOS in a healthy population [29] or individuals with chronic traumatic brain injury. However, Harro et al. [30] have reported that the Neurocom LOS is a valid and reliable measure of balance impairment in individuals with Parkinson Disease. Additionally, Navalón et al. [31] found calculated LOS composite scores to be a reliable and valid measure for detecting postural instability in individuals with acquired brain injury using the NedSVE/IBV system.

#### Cardiovascular

Cardiovascular fitness measures included resting heart rate (RHR) and heart rate at the end (HRe) of each mini-game. RHR was recorded after the participant had been lying supine for 15 min. HR was taken by a Polaris heart rate monitor and manually each time. The participant’s HR was monitored continuously throughout the intervention session using the Polaris heart strap and wristwatch monitor. The HRe was recorded as the HR at the end of each mini-game. The total activity time (TAT) constituted the total time that the participant was engaged with VR gaming. The time in training range (TTR) was the time the individual spent at a therapeutic heart rate.

### Intervention

A pre-intervention modified Balk-Ware maximal exertion treadmill stress test was used to determine the appropriate exercise intensity and establish the participant’s maximum HR. A HR above 40% heart rate reserve (HRR) based on the Karvonen method was considered therapeutic. This HR is commonly considered the threshold of moderate cardiovascular exercise [32]. Therapeutic heart rate was not altered throughout the duration of the intervention.

The participant completed 15 sessions of supervised VR training over an eight-week period. VR interventions were conducted in a private room within the outpatient Physical Medicine and Rehabilitation service to decrease distractions and provide adequate space for intervention. An Xbox One® and Kinect® sensor were used with a 45” Samsung television. The TV was placed approximately 8–10 ft away from participant situated at chest height. As needed, a therapist guarded the participant from behind to avoid motion sensor interference. The supervising therapists conducting the intervention have had 5 years of combined experience treating Veterans with TBI and 1 year of using Kinect games for physical therapy treatment.

Each session lasted between 50 and 60 min in total. A ten-minute period prior to the start of the intervention was used by the therapist to allow time for set-up of the gaming system and television. The intervention utilized two commercially available Xbox One Kinect games called “Shape Up” and “Kinect Sports: Rivals” to challenge both cardiovascular and balance systems. Each game is composed of mini-games (MG). Each game has a selection of MGs that vary in the type of challenge and motor pattern tasks. A pool of mini-games was selected to address domains of dynamic balance, static balance, and cardiovascular fitness that were appropriate for the participant based on clinical judgement. All MGs required the integration of visual and vestibular information to produce full body movements while maintaining postural stability and movement accuracy. Additionally, the intervention games used a closed-loop feedback system in which the games progressively challenged the participant based on their performance. Throughout the intervention the participant was allowed to give a preference toward the allowed MGs which he found the most engaging and enjoyable. Each MG lasted between 1:30 min to 4:00 min. Both games were played for approximately 25 min during each session. The first 10 min of MGs were used to warm-up. Rest breaks were allowed as the participant required them. A 5-min break at the 25^th^ minute of intervention was incorporated to allow for the change of the Xbox console game. The therapist was also able to implement environmental challenges for further progression. A MG that required maximal or total assistance to prevent a loss of balance was withheld for the remainder of the session. The MG could then be revisited during the next session. The name, summary, and progression of each mini-game used are listed in Table 3.

**Table 3:** Description and potential progression of supervised game play during clinical VR intervention

Mini-(Game)	Game Objective	Areas Challenged	Level Progression	Environmental Progression
Arctic Punch (SU)	Requires participant to punch across the body to hit targets	Cardiovascular endurance, postural control within stationary BOS, UE/LE movement speed/accuracy	Increase movement speed	Stand feet together, one foot, or on compliant surface
Knee Up Splash (SU)	Requires participant to break blue, green, and red watermelons with high knee motion in a predetermined order	Cardiovascular endurance, dynamic postural control, UE/LE movement speed/accuracy, memory	Increase length of colors to memorize based on performance, Increase movement speed	Perform on compliant surface
Squat Me to the Moon (SU)	Requires participant to perform bodyweight squats emphasizing depth	LE anaerobic endurance, postural control within stationary BOS, LE movement speed/accuracy	Increase movement speed	Perform on compliant surface
Stunt Run (SU)	Requires participant to run in place and jump, duck, or dodge left or right to avoid random obstacles	Cardiovascular endurance, dynamic postural control, LE movement speed/accuracy, reaction time	Obstacles occur more frequently based on performance	N/A
To the Core (SU)	Requires participant to rotate trunk left and right with shoulders and elbows flex to 90°	Abdominal anaerobic endurance, postural control within stationary BOS, abdominal movement speed/accuracy	Increase movement speed	Stand feet together, one foot, or on compliant surface
Tennis (KS)	Requires participant to perform overhead serve, forehand, and backhand rebound against computer opponent	Dynamic postural control, visual tracking, UE/LE movement speed/accuracy, reaction time	Requires maintained accuracy at increased speed based on performance	N/A
Rock climbing (KS)	Requires participant to reach and grab overhead and to sides in race against computer opponent	Postural control within stationary BOS, UE movement speed/accuracy	Requires maintained accuracy at increased speed based on performance	Perform on compliant surface

*SU* Shape-up, *KS* Kinect Sports, *BOS* base of support, *UE* upper extremity, *LE* lower extremity, *N/A* not applicable

### Data analysis

Statistical tests were performed using SPSS and the SINGWIN program. SINGWIN provides statistical testing for single system designs using guidelines as outlined by Bloom et al. [33]. Analysis of measures utilizing a celeration-line technique across consecutive phases was performed in SINGWIN. Within-phase data were evaluated using both a visual review of scatter plot data and by analysis via linear regression. Comparisons were performed between baseline and intervention phases as well as intervention and retention phases. Celeration lines were developed by first finding average of the first half of the phase’s data and the average of the second half. The respective averages were plotted at the 1^st^ and 4^th^ quarters along the x-axis, respectively. The two averages were then connected. The celeration line is then extrapolated into the following phase for analysis. If a within-phase trend was present, a Fisher’s Exact Test was used to compare data between consecutive phases. If no within-phase trend was present, a two-standard deviation Chi-square test for proportions was utilized. Due to the conservative nature of the two-standard deviation Chi-square test and inability to visually report all data with trends, Cohen’s *d* was used to further characterize changes between phases. Heart rate and activity time data were analyzed by dividing the intervention phase into 3 sub-phases. Each sub-phase consisted of 5 interventions. One-way ANOVA and post hoc Tukey-Kramer tests were used to evaluate differences. An α-level of .05 was used to determine statistical significance.

## Results

### Balance

Table 2 and 4 display the results for clinical measures of dynamic and static balance.

**Table 4:** Clinical balance outcome results of chi-square analysis and corresponding effect sizes across study phases for clinical balance outcomes

	Baseline - Intervention		Intervention - Retention	
	*p*-value	*d* (%)	*p*-value	*d* (%)
FRT				
Both	0.04	0.00 (0.0)	0.11	-1.07 (-36.0)
Right	1.00	0.50 (19.1)	0.11	-1.51 (-43.0)
Left	1.00	-0.17 (-6.7)	0.29	-0.66 (-25.0)
DGI				
	0.001	2.95 (49.8)	0.03	1.23 (39.0)

*d,* Cohen’s *d* effect size, *FRT* function reach test, *DGI* dynamic gait index

**Fig. 1:**
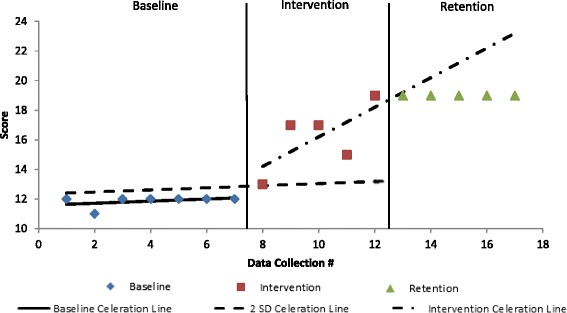
Dynamic gait index (DGI) scores across phases with celeration line analyses. Two-standard deviation (2 SD) celeration line was used for chi-square analysis between baseline and intervention phases as no trend present in baseline phase. The celeration line was carried through the retention phase for Chi-square analysis due to presence of upward trend in intervention phase

The participant exhibited significant improvements in dynamic balance, but measures of static balance were unchanged. The participant’s dynamic balance, as assessed by his DGI performance, showed a significant increase during the intervention and then plateaued during the retention phase as shown in Fig. 1. His ability to shift his COG forward, based on his FRTb values, demonstrated a significant reflection of the negative baseline trend during the intervention phase, *χ* ^2^ (1, *N =* 12) = 4.28, *p* = .04. During the retention phase the FRTb then returned to a negative trend, *χ* ^2^ (1, *N =* 10) = 4.29, *p* = .04.

Table 5 displays results of the LOS assessments.

**Table 5:** NeuroCom limits of stability results of chi-square analysis and corresponding effect sizes across study phases

	Baseline - Intervention		Intervention - Retention	
	*p*-value	*d* (%)	*p*-value	*d* (%)
EPE				
Front	1.00	0.29 (11.4)	1.00	1.18 (38.8)
Right	0.04	0.16 (6.4)	0.11	-0.07 (-2.8)
Left	0.07	0.48 (18.4)	1.00	-0.59 (-22.2)
Back	0.02	1.14 (37.3)	0.01	-0.57 (-21.6)
MXE				
Front	1.00	0.17 (6.7)	0.29	1.57 (44.2)
Right	1.00	0.30 (11.8)	0.04	-0.36 (-14.1)
Left	0.38	0.25 (9.9)	1.00	0.71 (26.1)
Back	0.79	1.09 (36.2)	0.11	-0.18 (-7.1)
DCL				
Front	0.22	1.27 (39.8)	0.04	-1.08 (-36.0)
Right	0.79	1.14 (37.3)	1.00	0.05 (1.9)
Left	0.22	1.19 (38.3)	0.11	-1.15 (-37.5)
Back	0.37	1.24 (39.3)	0.29	0.19 (7.5)

*d,* Cohen’s *d* effect size, *EPE* end-point excursion, *MXE* maximal excursion, *DCL* directional control

The LOS measures of postural control (EPE and MXE) showed inconsistent improvements and trend changes across phases while the measure of motor control (DCL) displayed notable, though not significant, improvements in all directions. During the intervention phase the EPE displayed a significant positive trend to the right, *χ* ^2^ (1, *N =* 12) = 4.28, *p* = .04, and a significant increase in the backward direction *χ* ^2^ (1, *N =* 12) = 5.6, *p* = .02. During the retention phase an increase was found toward the front *χ* ^2^ (1, *N =* 10) = 4.29, *p* = .04 and a significant negative trend was displayed in the backward direction *χ* ^2^ (1, *N =* 10) = 4.28, *p* = .04. The MXE displayed a positive trend in the intervention followed by a plateau during the retention in the right direction, *χ* ^2^ (1, *N =* 10) = 4.28, *p* = .04. The DCL demonstrated non-significant increases during the intervention in the forward (*d* = 1.27, 39.8%), backward (*d* =1.24, 39.3%), right (*d* =1.14, 37.3%), and left (*d* =1.19, 38.3%) directions. In the retention phase the DCL displayed a significant negative trend only in the forward direction, *χ* ^2^ (1, *N =* 10) = 4.28, *p* = .04.

### Cardiovascular

Cardiovascular and activity results are reported in Table 6.

**Table 6:** Heart rate and activity results of ANOVA and post-hoc testing across intervention sub-phase

	*F*	*p*-value	x (SD)
TAT	6.74	.01	
Sub-phase 1		*	27.15 (2.59)
Sub-phase 2			32.86 (1.91)
Sub-phase 3			30.50 (2.82)
TTR	4.19	.04	
Sub-phase 1		^	48.88 (7.68)
Sub-phase 2		^	51.21 (3.67)
Sub-phase 3			38.33 (7.80)
HRe	29.65	< .001	
Sub-phase 1		* ^	109.87 (15.74)
Sub-phase 2		^	102.40 (10.08)
Sub-phase 3			93.10 (8.86)

* - indicates significant difference from sub-phase 2. ^ - indicates significant difference from sub-phase 3. *TAT* total activity time, *TTR* time at therapeutic heart rate, *HRe* exercise heart rate at end of each mini-game

**Fig. 2:**
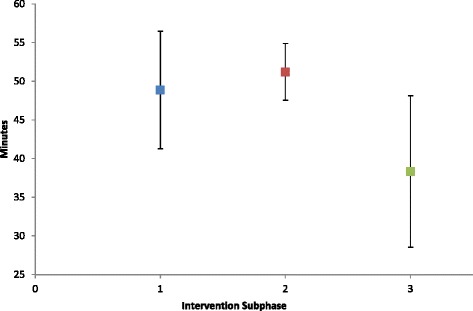
Time spent in therapeutic heart rate range Average time spent at heart rate greater than 40% of heart rate reserve during the 60 min treatment session across Intervention sub-phases

**Fig. 3:**
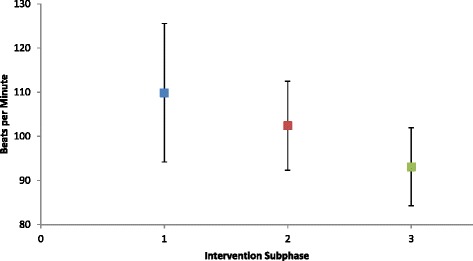
Mean heart rate at end of mini-game (HRe) Average exercise heart rate at the end of each mini-game (HRe) across Intervention sub-phases

The participant exhibited decreasing exertion, measured by HRe and TTR, alongside TAT that increased during the first 5 interventions and was maintained thereafter. One-way ANOVA between intervention sub-phases demonstrated a significant effect for TAT [*F* (2, 12) = 6.74, *p* = 0.01], TTR [*F* (2, 12) = 4.19, *p* = 0.04], and HRe [*F* (2,174) = 29.65, *p* = < 0.001]. TTR post-hoc Tukey HSD showed a significant difference between sub-phase 1 and sub-phase 3 as well as sub-phase 2 and sub-phase 3 as shown in Fig. 2. Post hoc Tukey-Kramer test showed that HRe was significantly different between all sub-phases as shown in Fig. 3.

## Discussion

In this proof-of-concept study, we developed and evaluated a rehabilitation program for an individual with chronic severe TBI using commercial Kinect-based VR games. To date, few studies have been published assessing the feasibility of using semi-immersive VR in persons with severe TBI [14, 34]. Our most notable finding in the current study was the participant’s improvement on the DGI, a measure of postural stability with gait, in comparison to those without, such as the FRT. This finding is in contrast to results by Llorens et al. [8, 10] where individuals with chronic stroke improved their postural control in non-gait tasks as assessed by the Berg Balance Scale after a rehabilitation intervention with the Xbox Kinect. However, this observation was in accord with improvements reported by other research groups exercising with commercial Kinect technology. For example, Song et al. [9] found greater improvements in gait speed and Timed Up and Go test times in individuals with chronic stroke who exercised using the Xbox Kinect instead of a bicycle ergometer. Similarly, Ilg et al. [11] reported significantly improved DGI scores of children with progressive degenerative ataxia after an intervention with commercial Kinect-based games.

Vestibular complaints such as dizziness, clumsiness and imbalance are frequent in veterans with all severities of TBI [35]. The DGI is a sensitive measure for fall risk in both the elderly and those with vestibular dysfunction [36, 37]. We hypothesize that the observed improvements were driven by the goal directed Kinect-based VR environment and MG selection which required the integration of visual and vestibular information. This assumption is supported by findings from Oritiz-Gutierrez et al. who found improved vestibular sensory integration outcomes in individuals with MS on the Neurocom Balance Master sensory organization test in response to a Kinect-based VR intervention.

We observed notable improvements in the accuracy of the participant’s COG moving toward a specified visual target (the DCL improved 37.3 to 39.8% for all directions) as reported from LOS testing. This finding suggested greater control of the COG during a dynamic movement task resulting in fewer deviations from the most efficient movement. We hypothesize that this finding represented a measure of improved motor control. The MGs require movement accuracy for success and, therefore, progressively challenge speed and accuracy of motor movements. We believe this was also instrumental to the observed DGI results through complementary sensory integration and motor improvements.

Unlike Ustinova et al. [14] we did not find a significant improvement in static balance based on the FRT scores. It is possible that our observed results were influenced by a potential ceiling effect. The participant’s MXE scores were within 5% of the expected range for his height and age during the baseline period. However, his baseline FRTl and FRTr scores were not significantly different from averages reported for Parkinson’s disease (Hoehn and Yahr stages 1–3) (mean (cm) = 33.54 (7.36)) [38] and peripheral vestibular disorders (mean (cm) = 31.7 (7.5)) [39]. Moreover, our intervention did not require progression through advancing static BOS exercises prior to beginning dynamic postural control exercises. It is also important to note that the Kinect-based intervention did not restrict the participant’s BOS in contrast to other VR interventions such as the Wii Fit board. Therefore, the participant may have resorted to a stepping strategy rather than challenge his postural control within a stationary BOS. We believe this also highlights a limitation of commercial games as therapists may have limited control over difficulty and intensity progression due to the game’s automated progression based on player performance.

However, use of game directed progression for intensity level did not appear to hinder cardiovascular adaptations for our participant. Our participant exhibited a significant decline in HRe and TTR, in parallel with increased and maintained TAT. Mossberg et al. [40] demonstrated similar changes in a population of individuals with acquired brain injury. Their participants completed physical therapy programs incorporating 15–20 min of moderate aerobic training 2–3 times per week. These investigators observed improvements in VO_2_ for any given workload without a change in peak VO_2_ and hypothesized that this was due to improved movement efficiency. Kinect-based gaming has been shown to provide moderate to vigorous cardiovascular exercise in healthy [41, 42], post-stroke, and cystic fibrosis [43] populations. Work by Sampaio et al. [20] found improved measures of HR variability to correlate with improved VO_2_ max in individuals post-stoke who reached ACSM guidelines for physical activity when exercising with commercial Xbox Kinect games. Although we did not measure HR variability or VO_2_ max as outcome measures, we suggest that the trend in our current study was also evidence of improved cardiovascular efficiency and that clinical measures of HR can provide a gross measure of exercise tolerance. Our program structure illustrated the feasibility of meeting guidelines for moderate aerobic training while using commercial Kinect games. These results warrant further study to investigate cardiovascular benefits of Kinect-based VR for the chronic TBI population.

The use of custom VR game designs undoubtedly can increase task specificity as well as therapist control of exercise intensity and progression. However, the clinical adoption of these laboratory developed games is significantly impeded in the near-term due to potential research, financial, marketing, and implementation challenges. Nevertheless, the advent of games designed specifically for rehabilitation purposes holds promise as their availability and utility increase over time. In our study, we successfully used commercial games in a supervised manner to improve balance in a veteran with a history of severe TBI. Additionally, we have provided descriptions of each game, appropriate impairment level targets and progressions for rehabilitation professionals. These are based on recommendations by Levac et al. [7] and the KWiC knowledge translation work. Further study is needed to improve the game selection criteria and implementation standards for various patient populations.

### Limitations

Limitations of our study methodology primarily relate to the nature of the single system design and evaluation of a young single participant who exhibited controlled hypertension and smoking history. Though the experimental design featured in this report provided considerable advantages over a case report due to use of repeated measures and statistical analyses, the generalizability of single system design studies is limited in comparison to other more conventional clinical research designs. Unequal data collection assessments occurred in each phase of the study secondary to scheduling challenges with the participant. While assessment intervals and frequency was not exact during each phase, we did manage to meet the accepted standard of 5 to 7 assessments per phase [44]. Finally, it is unknown how specific demographic characteristics affected the participant’s ability and willingness to engage in a VR intervention. For example, the participant may have had greater engagement using VR due to their age and gender. It is also unknown how the combination of clinical characteristics such as the severity of balance impairment, time since injury, presence of cardiac conditions, and smoking habits affected the participant’s ability to participate or reach moderate and vigorous exercise intensities during each MG. Replication and larger study designs using this intervention are required to improve the generalizability of the current findings.

## Conclusions

Based on our proof-of-concept study, a supervised Kinect based program using commercial games shows promise for improving balance dysfunction post severe TBI. However, our findings suggest that the use of commercial games may be more appropriate for young individuals with primary limitations in dynamic functional gait tasks rather than static postural control deficits. Preliminary evidence of this treatment protocol shows moderate cardiovascular exercise can be achieved. Our evidence also suggests cardiovascular adaptations occurred and should be further explored in an independent study. Further studies should include outcomes related to sensory organization and motor control to better understand the specific post-intervention adaptations that are augmented by VR-based training as well.

Finally, the present study provides additional support for the integration of Kinect-based commercial games into the clinical space as an adjunct or alternative to standard balance protocols for individuals with chronic TBI. Commercial games can likely enter the clinical space significantly sooner than custom lab-developed games and at a significantly reduced price. However, clinical judgement and criteria regarding game selection requires standardization based on individual abilities and limitations.

## Abbreviations

COG: Center of gravity
DC: District of Columbia
DCL: Directional control
DCVAMC: DC VA medical center
DGI: Dynamic gait index
EPE: End-point excursion
FRT: Functional reach test
GCS: Glascow coma scale
HHR: Heart rate reserve
HRe: Heart rate at end of mini-game
LOS: Limits of stability
MG: Mini game
MXE: Maximum excursion
RHR: Resting heart rate
TAT: Total activity time
TBI: traumatic brain injury
TTR: Time in training range
VA: Veterans affairs
VL: Maximal velocity
VR: Virtual reality
